# Partial Reproducibility and Pleiotropic Epigenetic QTLs in *Arabidopsis* Recombinant Inbred Lines

**DOI:** 10.3390/plants15152379

**Published:** 2026-08-03

**Authors:** Raul A. Faburrieta, Brenda A. López Ruiz, Ulises Rosas, Kenneth J. Davis, Christina L. Richards, Joshua A. Banta

**Affiliations:** 1 Department of Biology and Center for Environment, Biodiversity and Conservation, University of Texas at Tyler, Tyler, TX 75799, USA; raulfaburrieta@gmail.com (R.A.F.); kdavis83@patriots.uttyler.edu (K.J.D.); 2 Jardín Botánico, Instituto de Biología, Universidad Nacional Autónoma de México, México City 04510, Mexico; brenda.lopez@st.ib.unam.mx (B.A.L.R.); urosas@ib.unam.mx (U.R.); 3 Department of Integrative Biology, University of South Florida, Tampa, FL 33620, USA; clr@usf.edu

**Keywords:** *Arabidopsis thaliana*, epigenetics, epigenetic stability, quantitative epigenetics, epigenetic variation, epigenetic Quantitative Trait Locus (epiQTL) mapping, epigenetic Recombinant Inbred Lines (epiRILs), flowering time, fruit number, rosette diameter, rosette leaf number, basal branch number, lateral branch number, Auxin Response Factors (ARFs)

## Abstract

Unlike conventional genetic polymorphisms, many induced epigenetic polymorphisms can be reset across generations, raising uncertainty about how consistently the same epigenetic loci and associated phenotypic effects can be recovered among independent studies. To address this problem, we designed our study specifically to maximize a lineage-matched, environmentally aligned cross-study comparability with the foundational work of Cortijo et al. by using seeds derived from the same epiRIL generation and grown under broadly similar environmental conditions. We mapped flowering time, as well as five non-flowering traits that had not previously been mapped in this epiRIL population: rosette diameter, basal branch number, lateral branch number, fruit number, and rosette leaf number. We detected significant epiQTLs for all traits except lateral branch number. We also reproduced a facsimile of the original computational pipeline. This design provides an approximate upper bound on expected reproducibility before additional generations of methylation resetting or divergence among seed stocks could substantially influence the results. We recovered two of Cortijo et al.’s previously reported epiQTLs, failed to recover another, and found a different one, with power analyses suggesting that the discrepancies may reflect statistical power. Within overlapping epiQTL intervals across the five traits that had significant intervals, we identified 69 candidate genes exhibiting gene body methylation. Overall, our results reproduced some but not all previous epiQTL signals when comparisons were made using closely matched source material under similar conditions.

## 1. Introduction

Heritable phenotypic variation can be underlain by genetic and non-genetic processes [1,2,3]. But far less is understood about heritable non-genetic effects on phenotypes and evolution than about genetic effects on phenotypes and evolution, highlighting a significant gap in our knowledge of how mechanisms like epigenetic modifications contribute to phenotypic diversity [3]. Research suggests that epigenetic mechanisms can account for heritable phenotypic variation [3,4,5], and can have different ramifications for evolution from DNA sequence-based mechanisms [3,6,7,8]. Epigenetic mechanisms include chemical modifications of chromatin or transcribed DNA that can influence gene activity and expression without changes in DNA sequence [4,9]. The most well-studied epigenetic mechanism is the methylation of cytosines within DNA sequences [10,11], although other mechanisms, such as histone modifications and a heterogeneous assortment of RNA regulatory systems [12], have also been uncovered and studied intensively [10].

Although several studies have suggested that heritable epigenetic differences can provide an alternative to genetically based inheritance [7,13,14,15,16,17,18], it is often impossible to isolate the contribution of epigenetic differences from that of DNA sequence differences. To address this problem, Johannes et al. [19] derived Recombinant Inbred Lines (RILs) of *Arabidopsis thaliana* from parental lines with few sequence differences but different methylation profiles (epiRILs). The lines segregate almost exclusively for differentially methylated regions (DMRs) that have been stably inherited for at least eight generations. Among these epiRILs, multiple epigenetic QTLs (epiQTL) accounted for 60–90% of the heritability in the timing of flowering on 3 different chromosomes [18]. These findings demonstrate that differentially methylated regions can be passed on from parents to offspring and may act as loci controlling phenotypic variation, allowing for an additional source of mutation and phenotypic variation at the population level.

Yet, controversy persists over the importance of epigenetic factors to heritable traits and evolution [3,20,21,22]. Cytosine methylation states can be reset, either before reaching the germ line or after transmission to the offspring [3,6,7,8,23], and epigenetic states themselves may exhibit imperfect stability and transmission fidelity [24], potentially weakening or altering epigenetic associations among individuals within the same generation [25]. Therefore, not all epigenetic variants endure in subsequent generations, and the heritability attributable to epigenetic variation may diminish. Furthermore, transgenerational epigenetic inheritance itself is controversial due to a lack of clarity about the mechanisms [26], as well as potential confounding by genetic and ecological factors [27]. On the other hand, epigenetic mechanisms can exacerbate evolutionary change by serving as a bridge, fostering the phenotypic changes that respond to selection until genetic changes happen later [28,29,30,31]. In this way, epigenetics may be an important player in evolution [3], including macroevolution [32,33]. To understand the importance of epigenetic mechanisms, such as the methylation polymorphisms in the epiRILs, long-term evolution experiments are required to track the stability and heritability of epigenetic effects and the resultant phenotypes [34,35,36,37,38,39,40,41,42,43,44]. One complication is that, unlike genetically based long-term evolution experiments, epigenetic modifications may be stochastic and short-lived. Even among sibling individuals derived from homozygous, selfed parents, epigenetic patterns may not be identical; the same experiment could yield different outcomes when repeated with a different sample of seeds from the same generation, even within the same epiRILs [45,46,47].

As a first step, we evaluated the stability of methylation-associated phenotypic effects by deliberately designing our study to closely replicate part of Cortijo et al.’s [18] work. Although several subsequent studies have reported similar epiQTLs to those found in Cortijo et al. [48,49,50,51] they relied on epiRILs of unknown or unspecified generation, or on derivative seed stocks that were hybridized in new combinations. Furthermore, the analytical pipelines were different. As a result, it remains unclear how consistent the original epiQTL signals are when the same material, generation, and analytical framework are used. To address this gap, we used seeds derived from the same epiRIL generation, maintained under broadly similar environmental conditions, and analyzed with an analytical pipeline nearly identical to that used by Cortijo et al. [18]. This design isolated the extent to which the reported epigenetic associations can be recovered from the same seeds of the same generation when key biological and experimental factors are held as constant as possible.

We also included traits not examined by Cortijo et al. [18], specifically rosette leaf number, rosette diameter, basal branch number, lateral branch number and fruit number. These traits are known to exhibit natural variation among *A. thaliana* individuals and often show developmental or physiological correlations with flowering time [52,53]. We sought to identify candidate genes underlying significant epiQTL intervals shared by the traits. By integrating genomic resources from the Ensembl Plants [54] and *Arabidopsis* 1001 Genomes [55] projects, we screened thousands of protein-coding and epigenetically relevant genes within the epiQTL intervals in common between traits. This allowed us to refine our understanding of which genes may be regulated epigenetically and contribute to pleiotropic trait variation. We examined the methylation profiles of these candidate genes in different methylation contexts (CG, CHG, CHH), providing insight into their regulatory potential.

In summary, our study had three primary aims. First, we assessed whether previously reported flowering time epiQTLs could be recovered using a particularly stringent lineage-matched, environmentally aligned cross-study comparison with key aspects of Cortijo et al. [18] using sibling seeds derived from the same epiRIL generation and grown under broadly similar environmental conditions. Second, we evaluated epigenetic pleiotropic effects across multiple traits. Third, we performed candidate gene analyses within significant shared epiQTL intervals to identify protein-coding and epigenetically regulated genes that may underlie coordinated trait variation. Together, these analyses allowed us to evaluate the consistency of methylation-associated phenotypic effects within a single generation, using a common source material and similar experimental design.

## 2. Results

We used epigenetic quantitative trait locus (epiQTL) mapping to examine developmental and reproductive traits in *Arabidopsis thaliana*. To determine whether we could recover previously identified epiQTLs and detect additional loci, we analyzed flowering time (FT) and several traits not examined by Cortijo et al. [18]: the number of basal inflorescences (BBN), fruit number (FN), rosette diameter (RD), and rosette leaf number (RLN). A small number of plants never flowered, died, or were destroyed before all measurements could be taken. Five replicate plants were initially grown for each epiRIL. Across the 123 epiRILs, the mean numbers of surviving plants per line were 4.98 for flowering time, 4.98 for rosette diameter, 4.73 for basal branch number, 4.73 for lateral branch number, and 4.64 for fruit number. Thus, the averages were generally close to the intended five replicate plants per line. Our analyses recovered flowering-time epiQTLs on chromosomes 1 and 4 that were similar to those reported previously by Cortijo et al. [18], while also identifying differences between the two datasets that were consistent with limited statistical power. Across traits, several epiQTL intervals overlapped, including regions associated with FT, BBN, RD, and FN, suggesting either pleiotropic effects or closely linked loci. Candidate-gene screening within the overlapping intervals identified several genes with potential roles in epigenetic regulation and plant development.

### 2.1. Comparison of Flowering Time epiQTLs and Post-Hoc Power Analysis

**Figure 1:**
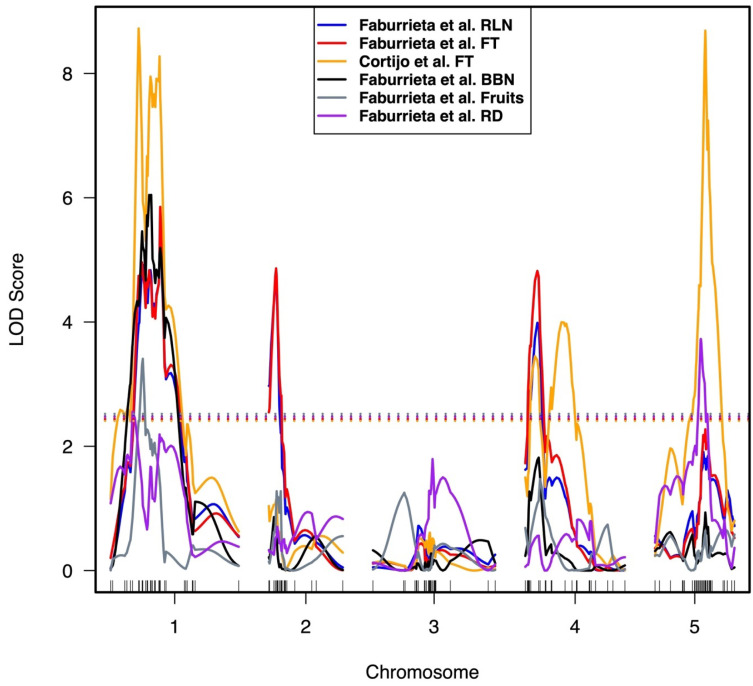
epiQTL mapping profiles for the following traits: flowering time (FT) under greenhouse conditions in the Cortijo et al. [18] study (Cortijo et al. FT) and under controlled growth chamber conditions in the current study (Faburrieta et al. FT), and rosette leaf number (RLN), basal branch number (BBN), fruit number (FT), and rosette diameter (RD) in the current study. The x-axis shows position on chromosome 1, 2, 3, 4, or 5 in centimorgans. The y-axis shows the LOD score, which for every marker is the log_10_ of the ratio of the probability that an epiQTL is present to the probability that an epiQTL is absent. The colored dotted horizontal lines show the independently calculated 5% genome-wide permutation threshold for each trait. Because the thresholds were similar, several lines overlap visually.

**Figure 2:**
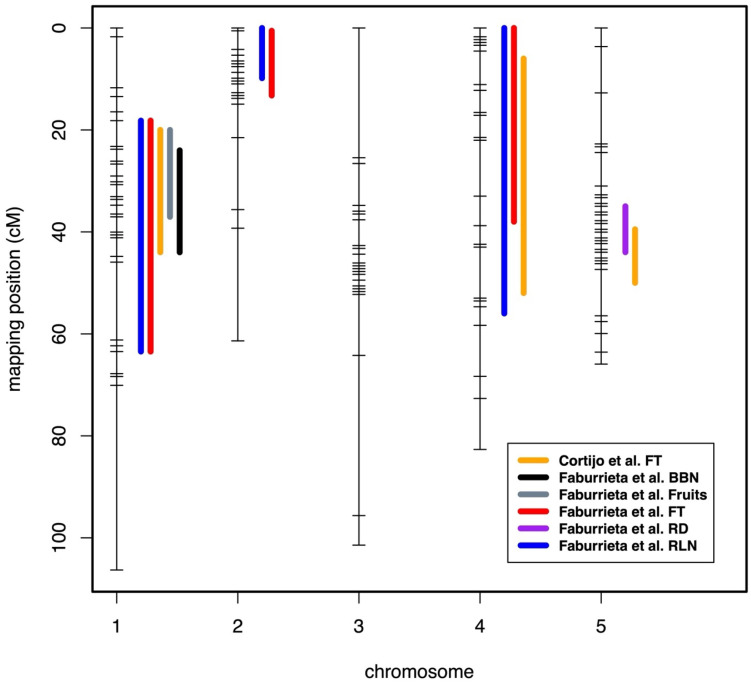
A genetic linkage map showing the 95% confidence intervals for the epiQTL regions in the current study and Cortijo et al. [18] for the indicated traits: flowering time (FT), rosette leaf number (RLN), rosette diameter (RD), the basal branch number (BBN) and fruit number (FN). The mapping positions of the epiQTL intervals are positioned according to the location on the chromosome in centimorgans.

We found epiQTLs on chromosome 1 and chromosome 4 that were similar to those identified by Cortijo et al. [18]. Additionally, our flowering time dataset had a significant epiQTL on chromosome 2 that was not present in the Cortijo et al. dataset. Conversely, Cortijo et al.’s flowering time dataset had an epiQTL on chromosome 5 that was not present in our dataset (Table 1; Figure 1 and Figure 2).

The power analysis indicated that the discrepancies between our results and those of Cortijo et al. [18] could be attributable to limited statistical power. For the chromosome 2 epiQTL detected in our dataset but not in the Cortijo et al. dataset, the estimated power of the Cortijo et al. dataset to detect the locus was low (0.055). The estimated effect size was also smaller in the Cortijo et al. dataset than in ours (0.47 versus 1.75; Table 2). Conversely, Cortijo et al. identified a significant epiQTL on chromosome 5 that was not significant in our dataset, although an elevated LOD score occurred at the same location in our analysis (Table 1 and Table 2; Figure 1 and Figure 2). The estimated power of our dataset to detect this epiQTL was moderate but insufficient (0.56), despite an estimated effect size of 1.09.

### 2.2. Overlapping epiQTL Intervals Across Multiple Traits

In our comparison of QTL intervals across multiple traits beyond just flowering time, we identified several regions of overlap. On chromosome 1, a single region was associated with FT, BBN, FN, and RLN, suggesting a potentially pleiotropic locus influencing reproductive timing, shoot architecture, and reproductive output. An alternative explanation is that the region contains a series of co-segregating loci, producing overlapping signals. On chromosome 2, we found overlapping QTLs for FT and RLN, while on chromosome 4, another shared interval was associated with both FT and RLN. Finally, an overlapping region on chromosome 5 was associated with FT and rosette diameter (RD), indicating a possible link between phenological and vegetative traits (Table 1 and Figure 1 and Figure 2). We found no epiQTL intervals associated with lateral branching.

### 2.3. Candidate Genes in the Overlapping epiQTL Intervals Are Linked to C Methylation and Auxin Signalling

We identified several genomic intervals with overlapping epiQTLs across traits and screened a total of 3408 protein-coding and epigenetically relevant genes located within these intervals. From this initial set, we narrowed the list to the most relevant candidate genes, filtering for those whose descriptions could be linked to the traits under study. This resulted in 93 candidate genes based on previous studies showing functional relevance to flowering time, rosette traits, branching, and fruit production. We further filtered to retain 69 genes exhibiting the highest gene body methylation in the three contexts, CG, CHG, or CHH in Col-0. We visualized these genes using bisulfite-sequencing data from leaf tissue [56].

**Figure 3:**
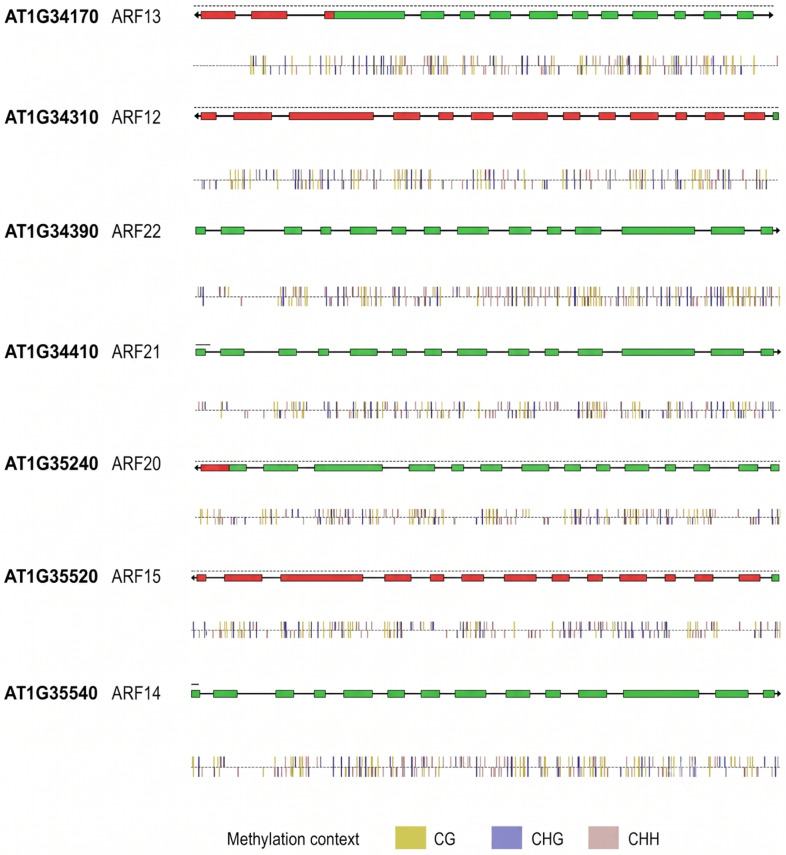
Methylation patterns in *ARF13*, *ARF12*, *ARF22*, *ARF21*, *ARF15*, and *ARF14* are localized within the epiQTL for flowering time, basal branch number, fruit number, and rosette leaf number. Large rectangles represent the exons, while the introns are shown as small grey rectangles. Below these, the methylation levels are indicated in three contexts: CG, CHG, and CHH using different colors. The red and green rectangles indicate strand and orientation information for transcripts mapped to opposite DNA strands. When red and green blocks appear next to each other, they show that the displayed genomic window includes strand-specific transcripts from both strands. Adapted from http://neomorph.salk.edu/1001.php, (accessed on 30 July 2026).

The candidate genes include multiple members of known regulatory families, such as Auxin Response Factors (ARFs), DNA methyltransferases, and chromatin remodeling factors. The ARF family of transcription factors is the central mediator of auxin-triggered transcriptional regulation, functioning from embryogenesis to flower development and seed set [57]. ARFs are potentially involved in the traits that we evaluated. Notably, seven ARFs—*ARF12*, *ARF13*, *ARF14*, *ARF15*, *ARF20*, *ARF21*, and *ARF22*—were located within epiQTL intervals shared by four traits: FT, RD, FN, and BBN. These genes exhibit high levels of methylation in the Col-0 wild type, particularly in the CG context. It is evident that in all the ARFs, the highest methylation levels in the wild type (Col-0) are found in the CG context, followed by the CHG context, and lastly, the CHH context. We also observed that methylation patterns were present in all the gene bodies of ARFs, including exons and introns, suggesting potential epigenetic regulation (Figure 3).

In addition, we identified candidate genes that control plant development at the epigenetic level, overlapped with our epiQTLs, and were methylated in the three contexts. Among these genes, we identified *DNA Methyltransferase 2 (DMT2)* within the QTL interval for flowering time and rosette leaf number (Table S2). Additional genes associated with epigenetic modifications include AT4G14000, a member of a putative methyltransferase family, and *MATERNAL EFFECT EMBRYO ARREST 57 (MEE57 or MET3)*, which is a DNA methyltransferase regulated by the Polycomb group complex during seed development [58]. Another gene was AT4G08990, an uncharacterized member of the DNA (cytosine-5-)-methyltransferase family, which is homologous to MET1. This gene is likely involved in the maintenance of CG methylation in the seed [59].

Furthermore, we identified the gene *MINICHROMOSOME MAINTENANCE 4* (*MCM4*) within the overlapping epiQTL intervals of RLN and FT on chromosome 2. This gene was linked to leaf surface area through genome-wide association studies (GWAS) [60]. Additionally, this gene was differentially methylated in the three contexts, depicting its potential to be controlled through epigenetic regulation (see Table S2).

## 3. Discussion

### 3.1. Partial Reproducibility of Flowering-Time epiQTLs

One of the central unresolved questions in epigenetics is not simply whether methylation-associated phenotypic effects can occur, but how consistently those effects can be recovered across repeated experiments and generations [3,24,61]. Unlike DNA sequence polymorphisms, epigenetic states may be incompletely transmitted, reset during development, or altered by stochastic processes [6,7,8], potentially weakening the long-term stability of epigenetically associated phenotypes [25,62]. Our study addressed this issue using a stringent, lineage-matched, environmentally aligned cross-study comparison with Cortijo et al. [18], employing sibling seeds from the same epiRIL generation, broadly similar environmental conditions, and a closely matched analytical framework. Under these conditions, we recovered some, but not all, previously reported flowering time epiQTLs, while also identifying additional loci. These findings suggest that at least some methylation-associated phenotypic effects can be partially reproducible within a single generation, while also highlighting that the stability and detectability of individual epiQTLs may vary across experiments even under closely matched conditions. However, although the comparison between our results and those of Cortijo et al. was lineage-matched and conducted under closely aligned environmental conditions, it was not a direct replication under identical conditions. Differences in laboratory environment, growth chambers, personnel, soil, watering, light, or other unmeasured factors may have influenced the observed phenotypes and methylation patterns. Consequently, differences between the studies cannot be attributed exclusively to changes within the epiRIL lines.

Importantly, the partial reproducibility we observed should not necessarily be interpreted as evidence against epigenetic contributions to phenotypic variation. Instead, our results may reflect the inherently probabilistic nature of epigenetic inheritance systems [24,63]. Even when using sibling seeds derived from highly selfed, near-isogenic lines, epigenetic states may diverge through imperfect maintenance, spontaneous epimutational processes, or environmentally sensitive methylation dynamics [25,62]. Consequently, epiQTL signals may vary in strength among replicate studies even under closely matched conditions. Our conditional cross-study power calculations also suggested that some non-recovered epiQTLs may have been difficult to detect given the sample sizes and residual variation in the respective experiments. Because these calculations used effect sizes estimated in one study rather than known true effect sizes, they should be interpreted as suggestive rather than definitive. Our study could not establish why any particular epiQTL was not recovered, but indicated that limited statistical power remains a plausible contributor to some of the discrepancies between studies. Thus, the observed differences may reflect a combination of biological instability, environmental variation, and sampling effects rather than the outright absence of epigenetic influence.

Our findings therefore support an intermediate perspective in the ongoing debate surrounding epigenetic inheritance [3,20,21]. The results do not support a model in which methylation-associated phenotypic effects are so transient that they are entirely irreproducible. Instead, they are more consistent with a system in which at least some epigenetic effects are recoverable across closely related experimental contexts but in which the magnitude and detectability of those effects may fluctuate even under closely matched biological and experimental conditions.

### 3.2. Overlapping epiQTLs and Potential Pleiotropy

An additional pattern emerging from our analyses was the occurrence of overlapping epiQTL intervals across multiple traits, suggesting that at least some methylation-associated loci may exert broader phenotypic effects rather than influencing traits independently. While other studies have identified pleiotropic effects within the epiRILs [48,49,50,51], the experimental conditions in those studies differed from those of Cortijo et al., and in some cases the plants were derived from the epiRILs but subsequently hybridized into new genetic combinations [50]. Consequently, direct comparisons among studies will not be readily interpretable. By using sibling seeds from the same documented epiRIL generation examined by Cortijo et al., our study minimizes this source of uncertainty and allows the degree of reproducibility to be interpreted within a shared generational context. In contrast, comparisons among studies in which the precise epiRIL generations are not documented are more difficult to interpret, because the extent of epigenetic divergence among the plant materials cannot be evaluated. Furthermore, environmental differences among studies may influence the expression of particular epiQTLs, such that epiQTL intervals identified under one set of growth conditions in one study may not reflect the same biological effects under another environmental context in a different study. For these reasons, we restrict our interpretation of pleiotropic effects to the present study and to Cortijo et al., representing a snapshot in time within a particular epiRIL generation and environmental context. Future comparisons using the same epiRILs in later generations may therefore help clarify how patterns of pleiotropy and epiQTL overlap change through time.

We identified epiQTL intervals on chromosomes 1, 2, 4, and 5 that were significantly associated with multiple traits, including FT, FN, BBN, and RD. The overlap among epiQTL regions is consistent with epigenetic pleiotropy and resembles classic genetic QTL findings in which a single locus affects multiple functionally related traits [64]. However, co-localization alone does not establish pleiotropy. Different genes or epigenetically modified loci within the same genomic region may instead influence different traits. Because methylation states at nearby loci can be correlated across chromosomal regions, overlapping epiQTLs could reflect either a shared causal epiallele or multiple linked epialleles with separate phenotypic effects [56]. Our mapping resolution does not allow these alternatives to be distinguished.

These possibilities have different functional and evolutionary implications. A shared causal epiallele could directly contribute to correlated variation among traits, whereas multiple nearby epialleles could produce a similar mapping pattern because they are inherited together. Finer-scale mapping and functional analyses of individual epi-loci would be required to distinguish pleiotropy from the effects of multiple linked epialleles [65,66,67].

### 3.3. Residual Genetic Variation in and Interpretation of epiQTL Effects

An important consideration in interpreting epiRIL studies is whether phenotypic differences attributed to inherited methylation states could instead reflect residual DNA sequence variation. The epiRIL population was designed specifically to minimize this possibility. The *ddm1-2* mutant had been repeatedly backcrossed into the Columbia background, the wild-type and mutant founders were near-isogenic, and only progeny homozygous for the functional *DDM1* allele were retained during construction of the epiRIL population. Thus, the lines were expected to segregate predominantly for methylation states inherited from the Col-WT and Col-*ddm1* founders rather than for widespread sequence polymorphisms.

The possibility of residual genetic variation was also examined empirically during the original characterization of the population. Johannes et al. [19] detected limited transposable-element movement, including some CACTA activity, but found no clear association between the observed insertion differences and flowering-time variation. Cortijo et al. [18] subsequently resequenced 52 of the 123 epiRILs and used targeted PCR in an additional 27 lines to investigate transposable-element insertions within the flowering-time and root-length epiQTL intervals. They identified four shared insertions, but the phenotypic effects were substantially weaker than those associated with the peak methylation markers, leading the authors to conclude that these insertions were unlikely to account for the mapped epiQTLs. Cortijo et al. also reproduced the association between methylation state and root length in a set of wild accessions that did not contain the identified shared insertions. While they did not to establish that the epiRILs are completely devoid of DNA sequence variation, and rare mutations or transposable-element insertions cannot be excluded as contributors to individual phenotype, the population design and the previous genetic analyses indicate that widespread segregating sequence variation is unlikely to be the primary explanation for the mapped effects. We therefore interpret the detected epiQTLs as predominantly associated with inherited methylation differences while recognizing that the distinction between genetic and epigenetic causation cannot be absolute without functional validation.

### 3.4. Candidate Genes Link Epigenetic Control to Developmental Networks

Our exploratory analysis identified 69 gene-body-methylated positional and functional candidate genes within epiQTL intervals shared by multiple traits. These genes were prioritized based on their genomic positions, annotated functions, previous trait associations, and the presence of gene-body methylation in Col-0. The resulting set should therefore be viewed as a hypothesis-generating list rather than as evidence that these genes are differentially methylated among the epiRILs or causally responsible for the mapped phenotypes.

### 3.5. Auxin Response Factors

Several auxin response factor genes were located within overlapping epiQTL intervals on chromosome 1, identifying auxin signaling as a plausible developmental pathway connecting flowering, branching, and reproductive traits. In particular, *ARF12* through *ARF22* have documented roles in embryo or endosperm development and are subject to chromatin-level regulation involving histone acetylation and deacetylation [68,69]. Their positions within shared epiQTL intervals, known developmental functions, and methylation in the wild-type background make them biologically plausible positional and functional candidates. These observations do not establish that the ARFs caused the mapped effects, but they provide a focused set of hypotheses for future investigation.

### 3.6. Additional Developmental and Epigenetic Candidate Genes

We also identified genes associated with epigenetic regulation or previously reported developmental phenotypes, including *DMT2*, *MET3* (*MEE57*), *AT4G08990*, and *MCM4*. *MEE57* is particularly noteworthy because it was previously proposed as a candidate gene involved in natural flowering-time variation in *A. thaliana*. In a preliminary analysis of natural accessions, methylation variation at a genomic region containing *MEE57* was associated with flowering time, with accessions carrying the methylated state flowering earlier than those carrying the unmethylated state [70]. Although that study could not exclude effects of linked genetic variation and did not establish causality, its findings provide independent support for considering *MEE57* a plausible candidate for further investigation. Evidence for the direct role of *DMT2* in DNA methylation remains limited [71], and it has not been described in the evaluated traits. *DMT2* is a gene involved in DNA methylation pathways that leads to a transcriptionally active chromatin state in the egg cell, similar to the chromatin configuration observed in apomixis [72]. However, reported *DMT2* expression in rosette and floral tissues makes it a plausible positional and functional candidate for rosette diameter and flowering time.

*MCM4*, which has previously been associated with leaf surface area in GWAS analyses [60], occurred within an interval shared by flowering time and rosette leaf number and exhibits CG methylation in Col-0. *MCM4* is crucial for ensuring proper DNA replication during the S phase of the mitotic cycle, which is essential for cell proliferation and overall plant growth. Additionally, loss-of-function mutants of this gene showed a reduction in effective leaf surface area [60]. These observations identify biologically plausible genes and pathways within the mapped intervals, but functional, expression, and methylation analyses are required to determine whether any of them contribute to the epiQTL effects.

## 4. Materials and Methods

### 4.1. Source of epiRILs, Experimental Conditions, and Measurements

We obtained the epiRILs used in Cortijo et al. [18] from the Versailles *Arabidopsis* Stock Center in September 2018. Colomé-Tatché et al. [73] and Cortijo et al. [18] used the same 123 lines to reveal segregating methylation polymorphisms that explained heritable effects for root length and flowering time. Johannes et al. [19] derived the epiRILs from two near-isogenic parental lines: the Columbia wild-type laboratory strain (Col-WT), and a mutant for the gene *DECREASED DNA METHYLATION 1* (*DDM1*, Col-*ddm1*, 4th generation) that exhibited 70% less methylation genome-wide than WT plants [19,74]. To do so, they backcrossed a single F1 individual to the Col-WT parental line, and then selected individuals that were homozygous for the *DDM1* wild-type allele for six generations. The epiRILs are nearly isogenic in terms of the DNA sequences and segregate mostly for DMRs, rather than DNA sequence-based polymorphisms [19].

The epiRILs include 126 DMRs that serve as markers, with an average spacing of 3.45 centimorgans (cM) (0.804 Mb) that covers 89.1 percent of the genome. We assumed the epigenotypes at each epilocus to be homozygous, due to the highly selfing mating system of *A. thaliana*. The epiRILs exhibit phenotypic variation and high heritability for traits related to growth and morphology, including plant height, flowering time, and primary root length [19,75].

We obtained seeds from the same generation as the core epiRIL material used for the epigenetic linkage map and the primary flowering time analysis reported by Cortijo et al. [18], as confirmed by Christine Camilleri of the Versailles *Arabidopsis* Stock Center (personal communication), namely the BC_1_-S_6_ generation, conventionally designated F_8_. We grew five replicate plants of each of the 123 different epiRILs for a total of 615 plants in a walk-in, controlled chamber at the University of Texas at Tyler. We used the same photoperiod and temperature conditions as Cortijo et al., which included 16 h day length with a temperature of 20–22 °C and an 8 h nighttime period with a temperature of 16–18 °C. Although we used the same epiRIL lines and reproduced the reported growth and phenotyping conditions as closely as possible, the experiments were conducted at different times, in different laboratories, and with different growth chambers and personnel. Therefore, unmeasured environmental and procedural differences may have remained between the studies.

In total, we measured six traits: flowering time (FT), rosette diameter (RD), basal branch number (BBN), lateral branch number (LBN), fruit number (FN), and rosette leaf number (RLN). We measured FT as the interval from planting to the first emergence of the primary flowering stalk, or inflorescence. At the same time that we recorded FT for each plant, we counted RLN, which is a well-established developmental proxy for the timing of the floral transition in *A. thaliana* [76,77], and measured RD, a commonly used proxy for maximum adult vegetative size [78]. At senescence, we counted BBN, defined as inflorescences emerging directly from the rosette; LBN, defined as secondary and higher-order branches arising from the basal inflorescences; and FN across all branches, which serves as an estimate of lifetime reproductive fitness [79].

### 4.2. epiQTL Mapping

Following Cortijo et al. [18], we performed single-epiQTL interval mapping, identically to standard single-QTL mapping, using Haley–Knott regression [80] with cytosine methylation variant markers (the loci were either homozygous for being methylated or homozygous for not being methylated) instead of DNA sequence markers (where the loci would typically be one homozygous genotype versus another homozygous genotype). We mapped the traits using the “scanone” function of the QTL package version 1.74 in R [81]. We utilized the same genetic map from the Cortijo et al. study. We empirically determined a separate genome-wide significance threshold for each trait using 1000 phenotype permutations. For each trait, we used the 95th percentile of the maximum genome-wide LOD scores from the permutation analysis as the significance threshold, corresponding to a genome-wide Type I error rate of 5%.

To calibrate our analysis pipeline, we re-analyzed the flowering time data from Cortijo et al., as their original pipeline was not publicly available. We applied the outlier criterion reported by Johannes et al. [19] for the original Julian flowering-date dataset later analyzed by Cortijo et al. [18]. Specifically, flowering-time values exceeding three standard deviations from the overall mean were excluded. Applying this criterion to the Cortijo et al. dataset resulted in the exclusion of two epiRILs. In our flowering-time dataset, we excluded one epiRIL as an extreme late-flowering outlier and two additional epiRILs because they germinated and reached the rosette stage but did not flower during the experiment. Thus, we excluded two epiRILs from the Cortijo et al. dataset and three from our dataset. The exclusions showed some consistency between studies: epiRIL60 was an extreme late-flowering outlier in both datasets, whereas epiRIL98 was an extreme late-flowering outlier in the Cortijo et al. dataset and did not flower in our experiment. We also applied the outlier criterion to the remaining traits for consistency, and excluded four epiRILs for basal inflorescence number, four for fruit number, four for lateral branch number, and two for rosette diameter.

### 4.3. Comparison of Flowering Time epiQTLs and Post-Hoc Power Analysis

We compared significant flowering-time epiQTLs identified in our dataset with those reported by Cortijo et al. [18]. Cortijo et al. evaluated only flowering time and primary root length, and primary root length was not measured in the present study. flowering time was therefore the only trait for which cross-study reproducibility could be assessed directly. Accordingly, post hoc power analyses related to reproducibility were restricted to flowering time. We mapped the remaining traits for the first time in this study and had no previously reported trait-specific epiQTLs against which reproducibility could be evaluated.

For each epiQTL that was significant in only one dataset, we used the effect estimated in that dataset as the effect size of interest and calculated the power of the other dataset to detect an effect of that magnitude. These calculations are conditional on the estimated effect sizes and were used as a cross-study sensitivity analysis. They should not be interpreted as definitive estimates of the true power or as proof that an undetected epiQTL was absent. We used the “powercalc” function of the qtlDesign package in R as a post-hoc analysis of the power to detect the epiQTLs. Following Cohen [82], we considered power values of 0.8 and higher to be sufficient to conclude that there was enough power to detect an effect of the size of interest. The effect size of interest corresponded to the effect size of the marker that had the highest logarithm of the odds (LOD) score within the epiQTL’s 95% confidence interval. According to Cohen [82], “a medium effect of 0.5 is visible to the naked eye of a careful observer. A small effect of 0.2 is noticeably smaller than medium but not so small as to be trivial. A large effect of 0.8 is the same distance above the medium as small is below it.” We used these conventions to determine if the effect sizes of the epiQTLs were small, medium, or large. To calculate power, we first calculated the environmental and genetic variances for the flowering-time phenotypes.

We also examined whether the 95% confidence intervals of significant epiQTLs overlapped across multiple traits in our dataset—specifically flowering time, basal branch number, fruit number, rosette diameter, and rosette leaf number—as well as flowering time in the Cortijo et al. dataset. Our aim was to identify shared genomic regions potentially harboring pleiotropic epigenetic mechanisms influencing multiple aspects of plant development and architecture.

### 4.4. Identification of Candidate Genes That Span the Intervals of Overlapping epiQTLs

We used the Bioconductor biomaRt R package to retrieve the athaliana_eg_gene dataset from Ensembl Plants (https://plants.ensembl.org/index.html; accessed on 1 February 2025), which provides genomic coordinates and functional annotations for *Arabidopsis thaliana* genes. For each of the five evaluated traits, we examined the previously reported epiQTL intervals and identified the shared lower and upper genomic boundaries of these intervals on each chromosome. We excluded Chromosome 3 because no epiQTL interval was identified on that chromosome. We then used the athaliana_eg_gene dataset to identify all genes located within these regions.

The epiQTL intervals contained 3408 genes (Table S1). We screened these genes using keywords (“methyl”, “flower”, “branch”, “meristem”, “shoot”, “auxin”, “cytokinin”, “seed”, “leaf”, and “gynoecium”) related to flowering date, rosette diameter, fruit number, basal branch number, and lateral branch number and retained protein-coding genes with known or putative functions related to one or more of these traits, as well as genes involved in epigenetic regulation (Table S2). We also searched the AraGWAS Catalog (https://aragwas.1001genomes.org; accessed on 1 March 2025) for genes within the epiQTL intervals that had previously been associated with these or related traits. This process identified 93 positional and functional candidate genes (Table S2).

We next used methylome data from the *Arabidopsis thaliana* 1001 Genomes collection (http://neomorph.salk.edu/1001.php; accessed on 1 March 2025) to determine whether these genes exhibit gene-body methylation in the Col-0 wild-type reference background. Examining Col-0 methylation provided a biologically motivated means of prioritizing genes because the epiRIL population was derived from a normally methylated Col-WT parent and a globally hypomethylated Col-*ddm1* parent. Thus, by design, the parental methylation polymorphisms that generated the epiRIL population were expected to occur primarily at sites that were methylated in Col-WT but lost or substantially reduced in methylation in the Col-*ddm1* parent. Genes that are methylated in Col-0 therefore represent plausible genomic locations at which *ddm1*-induced hypomethylated epialleles could have originated and subsequently segregated among the epiRILs.

We examined gene-body methylation in the CG, CHG, and CHH sequence contexts, where H represents A, C, or T. Genes without detectable gene-body methylation in Col-0 were excluded, leaving 69 gene-body-methylated positional and functional candidate genes for further examination (Table S2). This analysis was intended as a hypothesis-generating prioritization step rather than a direct test of methylation segregation or causality. Although methylation in Col-0 establishes that methylation could have been lost in the *ddm1* mutant lineage, it does not demonstrate that methylation at a particular gene differed between the parental lines, remained polymorphic among the epiRILs, cosegregated with the corresponding epiQTL, or caused the observed phenotypic variation. Follow-up studies would therefore be needed to compare methylation at these genes between the Col-WT and Col-*ddm1* parental lines, determine whether alternative methylation states segregate among the individual epiRILs, test whether those states are associated with both gene expression and the corresponding phenotype, and experimentally manipulate methylation at the strongest candidate loci to establish causality.

Finally, we reviewed the published literature and used the ePlant portal for plant genomic data and analysis tools (https://bar.utoronto.ca/eplant/; accessed on 1 May 2025) to investigate the expression patterns and known functions of the retained genes. We visualized methylated positions within their gene bodies using the physical methylome maps available from the Salk Institute 1001 Epigenomes Project website (http://signal.salk.edu/1001.php; (accessed on 30 July 2026)).

## 5. Conclusions and Future Directions

In conclusion, our study demonstrates that methylation-associated phenotypic effects in the *Arabidopsis* epiRIL system can be partially reproducibly recovered when biological material, environmental conditions, and analytical procedures are closely matched. By using sibling seeds from the same documented epiRIL generation examined by Cortijo et al. [18], we established a stringent framework for evaluating the reproducibility of epiQTLs within a shared generational context. Our results support a model in which at least some epiQTLs are reproducible within generations, while remaining sensitive to stochastic epigenetic processes, environmental conditions, and statistical power. The occurrence of overlapping epiQTL intervals across multiple traits, together with the identification of candidate genes associated with auxin signaling, methylation, and developmental regulation, further suggests that epigenetic variation may contribute to coordinated trait syndromes through pleiotropic or regionally linked mechanisms. Moving forward, fine-mapping [83], functional validation via epigenome editing [84,85], and transcriptomic profiling under variable environmental conditions [86] will be critical. Longitudinal studies tracking methylation states across generations and ecotypes will further elucidate the durability and ecological relevance of these effects [56,62]. Ultimately, incorporating epigenetic data into evolutionary and ecological models promises a richer and more complete understanding of the forces shaping phenotypic diversity. Considering the potential pleiotropic effects of epigenetic mechanisms is especially important for addressing longstanding questions in biology about the number and effect sizes of loci involved in evolutionary change [3,87].

## Figures and Tables

**Table 1:** The significant epiQTL markers found in our dataset and the Cortijo et al. [18] dataset, showing the point estimate of the most significant marker as well as the positions of the 95% confidence interval for the significant association. The mapping study from which the results were derived is indicated (either our dataset or Cortijo et al. [18] dataset), as is the phenotype that was mapped.

		Significant epiQTL Regions			
			Position (cM)		
Data Source	Phenotype	Chromosome	95% CI		
Faburrieta et al.	Flowering Time	1	18.18	41.15	63.46
Faburrieta et al.	Flowering Time	2	0.56	5.34	13.25
Faburrieta et al.	Flowering Time	4	0.00	10.00	38.00
Faburrieta et al.	Basal Branch Number	1	24.00	33.63	44.00
Faburrieta et al.	Fruit Number	1	20.00	26.69	37.05
Faburrieta et al.	Rosette Diameter	5	34.97	38.00	43.98
Cortijo et al. [18]	Flowering Time	1	20.00	40.59	44.00
Cortijo et al. [18]	Flowering Time	4	6.00	34.00	52.00
Cortijo et al. [18]	Flowering Time	5	39.49	41.73	50.00

**Table 2:** Post-hoc power analysis of epiQTL regions that were significant in one study [18] but not the other one. The table shows the power to detect an effect in the study where the effect was found versus the power to detect an effect in the study where the effect was not found. The maximal marker effect size within the significant epiQTL interval was used to calculate the power to detect an effect in that epiQTL interval. Presented are the positions of the markers with the highest LOD scores within the epiQTL regions, the number of distinct epiRILs that were used, and the number of replicates of each epiRIL that were used. The table also presents the effect sizes, the percent of the phenotypic variance explained by the significant epi-marker, the power to detect an effect of that size, and the LOD score of the maximal marker in that epiQTL interval, as well as the threshold that was used to determine significance (where above that threshold indicates significance).

Study	Chromosome	Position	*n*	Replicates	Effect Size	% Variance	Power	Threshold	LOD
Faburrieta et al.	2	5.34	120	5	1.75	20.61	0.99	2.34	4.73
Cortijo et al. [18]	2	6.47	121	6	0.66	6.1	0.28	2.46	1.14
Faburrieta et al.	5	41.73	120	5	1.09	9.18	0.56	2.49	2.27
Cortijo et al. [18]	5	41.3	121	6	1.51	25.11	0.99	2.41	8.69

## Data Availability

The primary data underlying these analyses have been deposited in https://github.com/RaulFaburrieta/The-stability-of-epigenetic-variants-associated-with-phenotypic-change.git, (accessed on 30 July 2026).

## Supplementary Materials

The following supporting information can be downloaded at https://doi.org/10.6084/m9.figshare.33137585 (accessed on 30 July 2026): Table S1: Genes located within the epiQTL intervals for the evaluated traits; Table S2: Positional and functional candidate genes within the epiQTL intervals, including gene-body methylation levels, annotated functions, expression patterns, and associated traits. Table S1 contains no literature citations. The references cited in Table S2 are included in the article reference list as References [58,59,60,69,88,89,90,91,92,93,94,95,96,97,98,99,100,101,102,103,104,105,106,107,108,109,110,111,112,113,114,115,116,117].
